# Editorial: Advances in the research of diabetic nephropathy, volume II

**DOI:** 10.3389/fendo.2023.1135265

**Published:** 2023-01-18

**Authors:** Katsumi Iizuka, Daisuke Yabe, Mohamed Abu-Farha, Jehad Abubaker, Fahd Al-Mulla

**Affiliations:** ^1^ Department of Clinical Nutrition, Fujita Health University, Toyoake, Japan; ^2^ Department of Diabetes, Endocrinology and Metabolism, Gifu University Graduate School of Medicine, Gifu, Japan; ^3^ Department of Rheumatology and Clinical Immunology, Gifu University Graduate School of Medicine, Gifu, Japan; ^4^ Center for One Medicine Innovative Research, Gifu University Institute for Advanced Study, Gifu, Japan; ^5^ Center for Healthcare Information Technology, Tokai National Higher Education and Research System, Nagoya, Japan; ^6^ Biochemistry and Molecular Biology Department, Dasman Diabetes Institute, Dasman, Kuwait; ^7^ Dasman Diabetes Institute, Dasman, Kuwait

**Keywords:** diabetic nephropathy, ciliogenesis, pyruvate kinase muscle type 2 (PKM2), miR-21, tubulointerstitial inflammation

In the past, renal impairment due to diabetes mellitus was predominantly a condition that was first preceded by albuminuria and then followed by a decrease in eGFR (1, 2). Recently, however, a decrease in glomerular filtration rate (GFR) without obvious albuminuria has been observed. Therefore, the concept of diabetic kidney disease has been proposed as an umbrella concept for diabetic nephropathy (1, 2). In recent years, improvements in renal prognosis with SGLT2 inhibitors and GLP-1 receptor agonists have been reported, and the effects of these drugs are actually felt in clinical practice (3), but the pathogenesis of DKD still remains largely unexplored.

Recently, the role of proximal renal tubule cells in the pathogenesis of diabetic kidney disease has been the focus of research (4). Diabetic glomerulonephropathy is mainly injury to glomeruli. In contrast, diabetic kidney disease involves not only renal glomerulonephropathy but also tubulointerstitial fibrosis. Based on the anatomically distinct regions of kidney biopsy samples, diabetic kidney disease can be divided into glomerular diabetic kidney disease and tubular diabetic kidney disease. The common characteristics of glomerular diabetic kidney disease involve a damaged glomerular filtration barrier, mesangial cell proliferation, and glomerulosclerosis. The major manifestations of tubular diabetic kidney disease include dysfunction of renal tubular reabsorption and secretion and tubulointerstitial fibrosis (4). The mechanisms of glomerular and tubulointerstitial injury in diabetic kidney disease are different, but there are many intersections of the related pathways and mediators. Furthermore, proximal tubular injury has an important role in the progression of diabetic kidney disease with/without proteinuria (2, 4). Therefore, in this volume, some papers focused on renal tubular epithelial cells.

The cilium is a microtubule-based organelle that projects from the surface of most vertebrate cell types and detects and transmits extracellular signals (5). The ciliary life cycle is also closely related to the cell cycle. Renal primary cilia are sensory antennas required for the maintenance of normal epithelial differentiation and proliferation in the kidney (5). In acute kidney injury, cilium length is increased at the early stage. In polycystic kidney disease, cilia are absent. Bai et al. focused on the role of renal primary cilia lengthening in the development of diabetic kidney disease (6). In humans, they showed that the number of ciliated cells and the length of cilia are positively correlated with the diabetic kidney disease class in the kidney biopsies of the patients with diabetic kidney disease. These results are consistent with those of STZ-injected mice and db/db mice (6). The researchers speculated that HDAC6-dependent tubulin deacetylation was inhibited in renal tubular epithelial cells in diabetic kidney disease (6). They also suggested that aberrant ciliogenesis may influence mitochondrial biogenesis and fatty acid β oxidation, accelerating renal fibrosis.

Pyruvate kinase muscle isozyme M2 (PKM2) forms both dimers and tetramers (7). In proliferation, the dimeric state of PKM2 promotes anabolism, such as in the polyol pathway, pentose phosphate pathway, and uronic acid pathway (7). In contrast, the tetrameric state of PKM2 promotes ATP synthesis and catabolism. The PKM2 activator TEPP-2 reduces PKM2 dimerization (7). Recently, TEPP-2 was shown to restore hyperglycemia-induced glomerular and tubular metabolic phenotypes, further inhibiting fibrotic progression in DKD. Wang et al. cultured HK-2 cells under sustained high glucose exposure (7 days) with (case group) or without (control group) the addition of TEPP-46 for another 1 day and analyzed the genome-wide transcriptome data from the case and control groups (7). Four extremely downregulated DE genes (HSPA8, HSPA2, HSPA1B, and ARRB1) and three extremely upregulated differentially expressed genes (GADD45A, IGFBP3, and SIAH1) are involved in decreased endocytosis (hsa04144) and the enhanced p53 signaling pathway (hsa04115), respectively. The researchers concluded that PKM2 tetramerization induced by TEPP-46 in hyperglycemic HK-2 cells reshaped the interplay among endocytic trafficking, dynamics of protein folding/unfolding, and the autophagy/lysosome system through the versatile networks of Hsp70s (7). Thus, these results partly provide a clue to resolve the mechanism by which PKM2 activators improve renal function. MicroRNA-21 regulates various biological functions, such as proliferation, differentiation, migration, and apoptosis (8). MicroRNA-21 is upregulated in the plasma, urine, and kidney tissues of patients with diabetic nephropathy and is anticipated to be a biomarker predicting the progression of diabetic kidney disease (8). Therefore, microRNA-21 plays an important role in the development and progression of DN (8). Liu et al. reviewed the progress of microRNA-21 in the pathogenesis of diabetic nephropathy (8). MicroRNA-21 exhibited its pathogenic roles in DN by forming a complex network with targeted genes such as MMP-9, Smad7, and TIMP3 and signaling cascades such as the Akt/TORC1 signaling axis, TGF-β/NF-kB signaling pathways, TGF-β/SMAD pathway, CADM1/STAT3 signaling, and AGE-RAGE regulatory cascade. In particular, miR21 suppression improves renal inflammation. For example, miRNA-21-5p suppression prevented macrophage infiltration, podocyte loss, and interstitial fibrosis and improves microalbuminemia (8). In rat renal tubular epithelial cells and mesenchymal cells, microRNA-21 suppressed Smad-7 expression and thereby enhanced the levels of TNF-a and Il-1b (8). Thus, the review showed that miRNA-21 has an important role in the development of diabetes kidney disease.

From another perspective, a study focusing on inflammatory cells infiltrating the kidney was also included as well as a study focusing on copper-related genes (9) and a study analyzing peripheral blood samples from diabetic nephropathy with the CyTOF test (10). In the former, biomarkers related to PTGS2, DUSP1, JUN, FOS, S100A8, S100A12, NAIP, CLEC4E, CXCR1 and CXCR2 were obtained; in the latter, biomarkers related to CTLA-4, CXCR3, PD-1, CD39, CCR4 and HLA-DR.

In conclusion, this special edition of “*Advances in the Research of Diabetic Nephropathy II*” provides new insights into the pathogenesis of diabetic kidney disease. A deeper understanding of the role of renal proximal tubule epithelial cells in renal fibrosis will be beneficial not only for finding new biomarkers for diabetic kidney disease but also for preventing the development of tubulointerstitial fibrosis and thereby diabetic kidney disease.

## Author contributions

KI wrote the first draft of the manuscript. KI, DY, MAB, JA and FA-M edited and revised the manuscript. All authors contributed to manuscript revision and read and approved the submitted version.
